# Drugs and Disease

**Published:** 1895-05-25

**Authors:** 


### Drugs and Disease.

Few forms of knowledge are so delusive as the crude physiology so usually taught and so commonly accepted by general readers as sufficient explanation of the mystery of life. Students, and even students of medicine, are far too apt to limit their conception of the possibilities and varieties of chemical change and interaction to the very narrow field of chemical knowledge which they are compelled to master at their schools; and although we would be the first to deprecate any rush to the other extreme, any indifference to exactitude or acceptance of the easy doctrine that the conditions of physiological chemistry are so complex that anything may happen, and that to "nature" nothing is impossible, yet the fact certainly must be accepted that no chemistry that we yet know, no science of weights, measures, and exactitude can yet explain a great many of the essential factors in some of the most common processes in physiology and pathology. Ultimately the action of both foods and drugs and poisons is a chemical action. Molecules are rearranged and energy is given off. With normal food this happens in a normal way, and the organism is meanwhile nourished; with drugs the action is abnormal, although sometimes this abnormality may correct one in an opposite sense caused by disease; with poisons the action is injurious. But in each case the substance taken into the body acts upon it by altering the chemical constitution of the media in which certain of its cells go through their daily work. Of what these changes consist, however, it is quite impossible at present to frame any accurate conception. The chemistry of muscle and of nerve, the changes which take place in action and in rest, the constant oscillation from one form to another among the infinite varieties of molecular construction which proteid bodies can take on, tries the intellect of the most skilled investigators, and utterly baffles the intelligence of the ordinary student. What, then, are we to think of the difference between the chemistry of one muscle and another, of one nerve and its neighbour, of one strand of fibres and others in the same bundle? And yet such differences must exist, for only by chemical affinities being satisfied in different degree by different nerves can we explain the selective action of certain poisons, which pick out certain tracks of nervous tissue and on them concentrate their injurious effects.

Lead is a striking instance of this, but many others might be quoted, and recent discoveries regarding the toxic substances developed during infectious fevers are likely to considerably enlarge the list available. Speaking recently on this subject at the National Hospital for the Paralysed and Epileptic, Dr. Gowers said, "You can conceive of no structural relation, or relation to vessels, which determines why lead acts upon the musculo-spiral nerve and not upon any other, and why it acts only upon some of the branches. It can only be due to some slight inherent difference in the minute constitution of the nerves which enables the atoms of lead to join with the atoms and molecules of these nerves and derange the process of their nutrition, acting on these nerves and not on others."

Lead, however, is only a single instance. It is every year becoming more clear how many diseases, and how many symptoms are due to what may be broadly spoken of as "toxic" agencies, the circulation in the animal fluids of abnormal and poisonous substances. Every process of life is due to the action of individual cells, but whether these act normally or abnormally, whether they maintain their nutrition or become degenerate, depends not wholly on their inborn tendencies, but upon the medium in which they live, and from which they draw their pabulum. This is a matter of chemistry, and it is obvious what an important bearing this fact has upon the treatment of disease by drugs and by diet. The more accurately we can express vital action in terms of chemical interaction, the more may we hope to fill up gaps and correct abnormalities in the chemical constitution of the animal fluids by adding to them the lacking elements or correcting their deficiencies by chemical means.

The wonderful physiological discoveries of recent years, especially those showing the extreme complexity of the organism, have tended to induce among many of our best men a sort of despair as to the possibility of useful interference with so delicate a mechanism. Side by side, however, with advances in physiology has been a widening of our knowledge of organic chemistry—and there lies our hope. So far as disease arises from chemical disturbances it should be amenable to chemical rectification.

